# 

**Published:** 1880-06

**Authors:** 


### CONTENTS FOR JUNE, 1880.

ORIGINAL COMMUNICATIONS.

ART. I.—Sexual Exhaustion (Neurasthenia). By GEORGE M. BEARD, M. D. .................. 271

ART. II.—Constitutional Origin of Diphtheria. By R. M. GRISWOLD, M. D. .................. 270

ART. III.—No "Battery" in a Tooth. By CHAS. MAYR, A. M. .................. 284

ART. IV.—Fistula. By G. HALSTED BOYLAND. 286

ART. V.—Treatment of Fracture of the Jaw, with Critical Remarks. By THOMAS BRIAN GUNNING, D. D. S. .................. 296

EDITORIAL DEPARTMENT.

Medical Care and Attention to the Indigent Sick.. 304

Some of our Medical Universities and Colleges. . 305

Pure Drinking Water.................. 306

Marine Hospital for Baltimore.................. 306

To Former Students.................. 307

The Prospective Advantages of Baltimore as a Medical Centre.................. 407

Hygiene.................. 308

Advertising Physicians.................. 308

Infidelity and Contempt of Religion.................. 309

Meetings of Dental Societies.................. 311

The Tenth Annual Session of the New Jersey State Dental Society.................. 311

BIBLIOGRAPHICAL DEPARTMENT.................. 312

HYMENEAL. NATALITIAL. NECROLOGICAL.... 314

EXCERPTA.................. 315

Salicylate of Potash.................. 315

United States Postal Law.................. 315

Mortuary Statistics and Meteorological Report... 316

